# Clinical Efficacy of Including Capecitabine in Neoadjuvant Chemotherapy for Breast Cancer: A Systematic Review and Meta-Analysis of Randomized Controlled Trials

**DOI:** 10.1371/journal.pone.0053403

**Published:** 2013-01-03

**Authors:** Qiuyun Li, Yi Jiang, Wei Wei, Huawei Yang, Jianlun Liu

**Affiliations:** Department of Breast Surgery, The Affiliated Tumor Hospital of Guangxi Medical University, Nanning, China; Sudbury Regional Hospital, Canada

## Abstract

**Background:**

Capecitabine has proven effective as a chemotherapy for metastatic breast cancer. Though several Phase II/III studies of capecitabine as neoadjuvant chemotherapy have been conducted, the results still remain inconsistent. Therefore, we performed a meta-analysis to obtain more precise understanding of the role of capecitabine in neoadjuvant chemotherapy for breast cancer patients.

**Methods:**

The electronic database PubMed and online abstracts from ASCO and SABCS were searched to identify randomized clinical trials comparing neoadjuvant chemotherapy with or without capecitabine in early/operable breast cancer patients without distant metastasis. Risk ratios were used to estimate the association between capecitabine in neoadjuvant chemotherapy and various efficacy outcomes. Fixed- or random-effect models were adopted to pool data in RevMan 5.1.

**Results:**

Five studies were included in the meta-analysis. Neoadjuvant use of capecitabine with anthracycline and/or taxane based therapy was not associated with significant improvement in clinical outcomes including: pathologic complete response in breast (pCR; RR = 1.10, 95% CI 0.87–1.40, p = 0.43), pCR in breast tumor and nodes (tnpCR RR = 0.99, 95% CI 0.83–1.18, p = 0.90), overall response rate (ORR; RR = 1.00, 95% CI 0.94–1.07, p = 0.93), or breast-conserving surgery (BCS; RR = 0.98, 95% CI 0.93–1.04, p = 0.49).

**Conclusions:**

Neoadjuvant treatment of breast cancer involving capecitabine did not significantly improve pCR, tnpCR, BCS or ORR. Thus adding capecitabine to neoadjuvant chemotherapy regimes is unlikely to improve outcomes in breast cancer patients without distant metastasis. Further research is required to establish the condition that capecitabine may be useful in breast cancer neoadjuvant chemotherapy.

## Introduction

Neoadjuvant therapy, also named preoperative therapy, was initially introduced to reduce the size or the extent of breast cancer in order to render the inoperable tumor operable, thereby permitting definitive surgery [1], [2]. While polychemotherapy involving anthracycline or taxane remains the most widely used neoadjuvant regimen, an increasing number of new chemotherapeutics are being tested in clinical studies of breast cancer neoadjuvant therapy.

Capecitabine is one such chemotherapeutic. It is an oral prodrug of 5′-deoxy-5-fluorouridine (5′-DFUR), which is effective and convenient, both as a monotherapy and as an addition to intravenous polychemotherapeutic to treat several types of cancers [3]. Furthermore, capecitabine works even better in combination with taxane because the latter shows synergistic effects with capecitabine [4]. Capecitabine is effective for salvage treatment of patients with metastatic breast cancer [5], [6], [7]. Therefore, the Breast Cancer Guidelines Committee of the National Comprehensive Cancer Network (NCCN) declared capecitabine/docetaxel to be a preferred combined chemotherapy regimen for recurrent or metastatic breast cancer [8].

Capecitabine has also been used in neoadjvant chemotherapy in clinical practice. Several Phase II clinical studies assessing capecitabine in neoadjuvant chemotherapy [9], [10], [11], [12], [13], and several randomized controlled trials examining neoadjuvant combinations containing capecitabine [14], [15], [16], [17], [18] have been carried out. However, the results of these studies have been controversial. Both the Austrian Breast and Colorectal Cancer Study Group Trial 24 trial (ABCSG 24) [18] and Keun et al trial [17] found that adding capecitabine to neoadjuvant therapy led to improvement in the pathologic complete response (pCR) of breast cancer patients. Whereas, the NSABPB-40 trial [16] and GEPARQUATTRO study [14] reported no significant difference in pCR between neoadjuvant therapy with and without capecitabine. In the ECTOII trial [15], pCR was more common in patients with estrogen receptor-negative (ER^−^) breast cancer than in patients with estrogen receptor-positive (ER^+^) breast cancer and capecitabine was only associated with a higher frequency of pCR only in ER^+^ cancer patients who received only AT-CMX neoadjuvant therapy. This study suggested that capecitabine might have efficacy as a neoadjuvant therapy under certain condition.

To update the results and clarify the uncertain efficacy of capecitabine in neoadjuvant treatment for breast cancer patients, we performed a meta-analysis of randomized controlled trials comparing neoadjuvant therapies with and without the drug for patients with breast cancer.

## Methods

### Publication Search

This meta-analysis was guided by the PRISMA (Preferred Reporting Items for Systematic Reviews and Meta-Analysis) Statement issued in 2009 (Checklist S1). The following databases were searched as recently as March 26, 2012. PubMed (1966 to present), online abstracts from the proceedings of Annual Meetings of the American Society of Clinical Oncology (ASCO 1992–2011) www.asco.org and online abstracts from the San Antonio Breast Cancer Symposium (SABCS 2004–2011) www.sabcs.org using the following keywords: “capecitabine” or “Xeloda” and “neoadjuvant or preoperative”, and the expanded MeSH term “breast neoplasms”. Manual searches were done by reviewing the reference lists of retrieved studies, textbooks and review articles to identify additional potentially eligible studies.

### Inclusion Criteria

To be considered eligible for inclusion in our meta-analysis, the study crieteria had to include:(a) a patient diagnosis of breast cancer without metastasis; (b) being a controlled trial; (c) using capecitabine in the neoadjuvant setting to treat breast cancer; (d) reporting relative risk (RR) with a 95% confidence interval (CI); if not, the reported data of outcomes pCR, BCS or ORR were sufficient to calculate them; and (e) being written in the English language.

Two authors (QYL and YJ) independently carried out literature searches and identified eligible articles based on the inclusion criteria. In cases of disagreement, a consensus was reached through discussion; when this did not work, a third author (JLL) independently came to a final decision. If multiple publications covering the same trial were retrieved, or if publications had overlapping study publications, only the most recent publication with the largest number of participants was included.

### Data Extraction and Quality Assessment

Data extraction and quality assessment were conducted independently by two authors (QYL and YJ). The following data from each eligible trial was extracted: authors' names, the journal, year of publication, trial design, patient eligibility criteria, baseline patient characteristics, dosing regimens, age range, clinical tumor stage, clinical node stage, end-points and the adverse events(AE’s) (NCI CTCAE Grades 3 to 4). Study quality was assessed using the Jadad score [19], which assessed the trials according to the following three questions: (1) does the study use an appropriate randomization method (0–2 scores)?; (2) does the study use an appropriate blinding method (0–2 scores)?; and (3) and, does the study report withdrawals and dropouts (0–1 scores)?.

### Statistical Analysis

The primary study end-point was the pCR in breast. Secondary endpoints were tnpCR, BCS and ORR. Outcomes measures were based on the intention-to-treat (ITT) analysis. The adverse events (AE’s) of neoadjuvant chemotherapy were analyzed using the NCI Common Terminology Criteria of Adverse Events 3.0 (NCI CTCAE Grades 3 to 4). RR and 95% CI were used to estimate the efficacy of capecitabine in neoadjuvant therapy for breast cancer. OR and 95% CI were used to estimate the drug-related toxicities. A heterogeneity assumption was calculated using the chi-squared-based Q-test (p<0.10 was considered significant) or the I^2^ statistic to examine the extent of between-study heterogeneity. Data were combined according to both the fixed-effect model (Mantel–Haenszel’s method [20]) and the random-effect model (DerSimonian and Laird’s method [21]).

Funnel plot and Egger’s test [22] were calculated to investigate potential publication bias. Sensitivity analyses were used to estimate the influence of individual studies on the overall effect. All statistical analyses were performed using RevMan 5.1. All P-values are two-sided.

## Results

### Description of Eligible Studies

**Figure 1:**
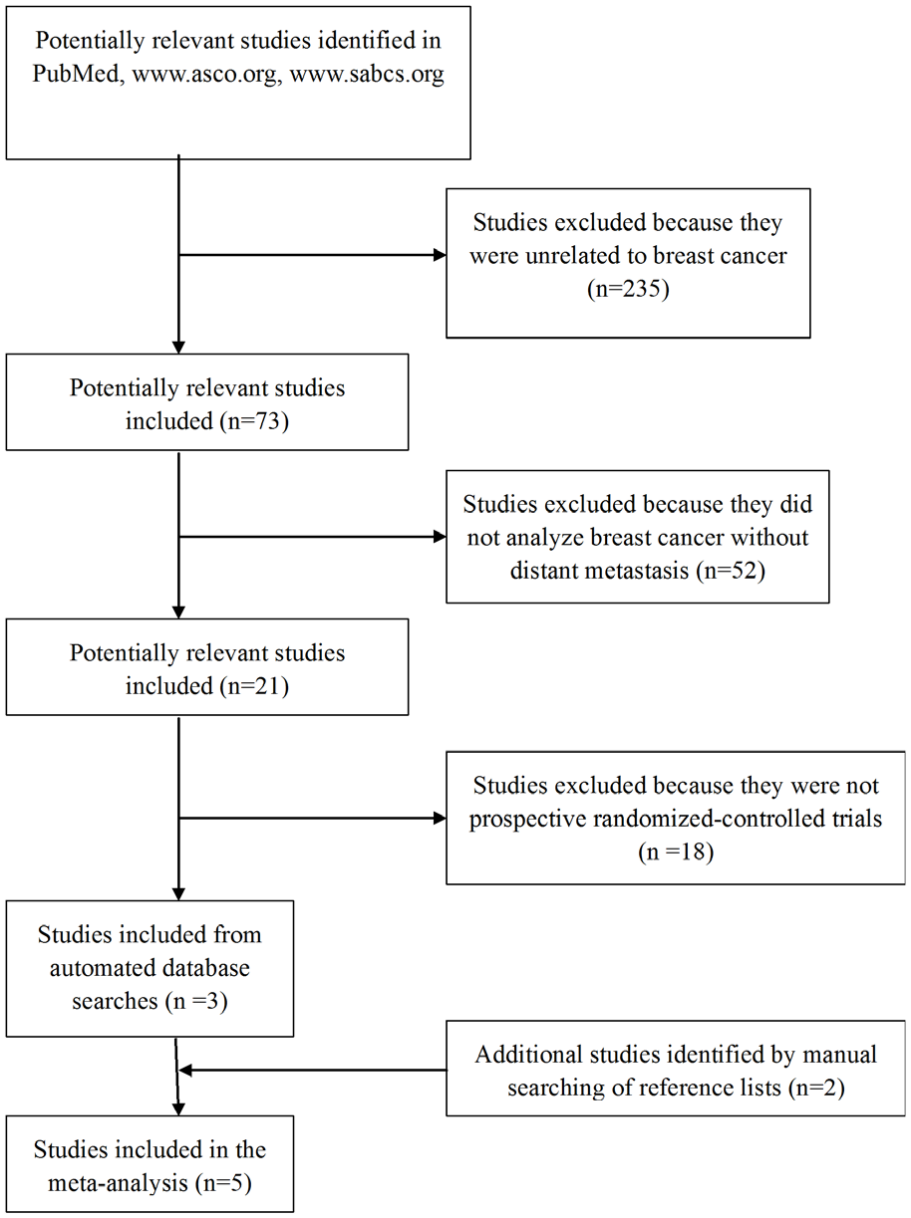
Flow chart of study selection.

A total of 5 trials [14], [15], [16], [17], [18] involving 3257 patients with early or operable breast cancer without distant metastasis were deemed to be eligible according to the inclusion criteria (Figure 1). The study characteristics were summarized in Table S1.

For the NSABP B-40 study [16], data were reported for a group receiving capecitabine as neoadjuvant therapy and for a group receiving gemcitabine. Data for the latter group were excluded from the meta-analysis based on the inclusion criteria. The ABCSG24 trial [18] was available only as an abstract, while full text was available for the remaining four trials. All five trials were open-label and randomized, involving concurrent or sequential use of anthracycline and/or taxane and capecitabine. The main endpoint in all included studies was pCR and secondary endpoints were tnpCR, BCS and ORR. Chemotherapy protocols of the included trials were listed in Table S1.

### Meta-analysis of the Primary Endpoint

**Figure 2:**
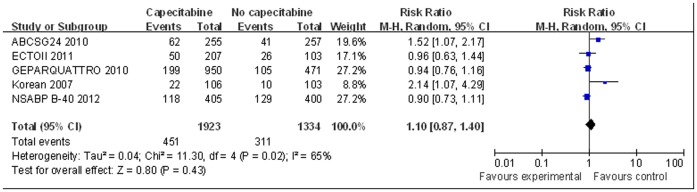
Forest plot to meta-analyze pCR outcomes for neoadjuvant therapy with or without capecitabine.

Neoadjuvant chemotherapy with capecitabine, anthracycline and/or taxane was not statistically significantly associated with improved pCR in patients with breast cancer compared with neoadjuvant chemotherapy usig anthracycline and/or taxane (pooled RR = 1.10, 95% CI 0.87–1.40, p = 0.43, random-effect model; Figure 2). There was significant heterogeneity among the studies for this outcome (I^2^ = 65%, p = 0.02).

### Meta-analysis of the Secondary Endpoints

**Figure 3:**
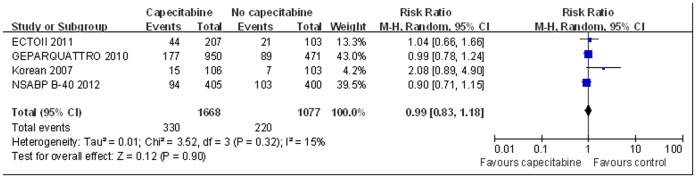
Forest plot to meta-analyze tnpCR outcomes for neoadjuvant therapy with or without capecitabine.

**Figure 4:**
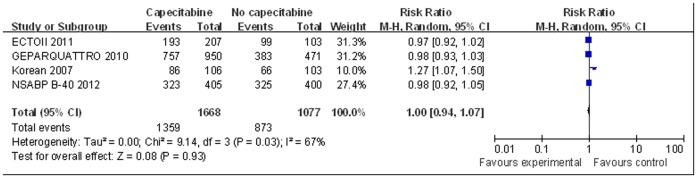
Forest plot to meta-analyze ORR for neoadjuvant therapy with or without capecitabine.

For meta-analysis of tnpCR and ORR, the ABCSG24 trial [18] was excluded because it did not provide necessary data. There was no significant difference in tnpCR between neoadjuvant therapy with and without capecitabine based on the fixed-effect model (RR = 0.99, 95% CI 0.83–1.18, p = 0.90, Figure 3). There was no significant heterogeneity among studies for this outcome (I^2^ = 15%, p = 0.32). Similarly, ORR was not significantly better for the patients receiving capecitabine as neoadjuvant therapy based on the random-effect model (RR = 1.00, 95% CI 0.94–1.07, p = 0.93, Figure 4). There was significant heterogeneity among the studies for this outcome (I^2^ = 67%, p = 0.03).

**Figure 5:**
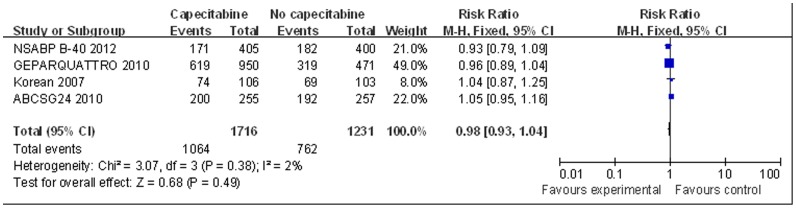
Forest plot to meta-analyze BCS outcomes for neoadjuvant therapy with or without capecitabine.

For meta-analysis of BCS, the ECTOII [15] study was excluded for lack of necessary data. Data pooled from the remaining four studies showed no significant difference in BCS between groups treated with or without capecitabine as neoadjuvant therapy, based on the fixed-effect model (RR = 0.98, 95% CI 0.93–1.04, p = 0.49, Figure 5). No significant heterogeneity was found among these studies for this outcome (I^2^ = 2%, p = 0.38).

### Toxicities

Data concerning AE’s were extracted from 3 trials [14], [16], [17]. A summary of drug-related toxicities ((NCI CTCAE Grades 3 to 4) is shown in Table S2. The pooled ORs of each group, stratified for toxicities greater than grade 3, indicated that a significant increase in toxicity was associated with neoadjuvant chemotherapy using capecitabine, anthracycline and/or taxane. Toxicities were identified as febrile neutropenia(OR = 1.54, 95% CI1.11–2.15 p = 0.01), hand-foot syndrome(OR = 7.26, 95% CI2.35–22.43, p<0.01). However, heterogeneity among trials was found in this analysis, possibly due to the use of different agents at various dosage and the use of different control arms.

### Publication Bias and Sensitivity Analysis

We performed funnel plot analysis and Egger's test to assess publication bias. This analysis showed no publication bias. As a result, there was no publication bias in each test (p = 0.109) for the primary endpoint analysis and for the secondary endpoint analysis (data not shown). Sensitivity analyses were used to determine the influence of individual studies on the overall RR estimates. No individual study affected the RR significantly for pCR, tnpCR, BCS, or ORR.

## Discussion

Capecitabine has proven effective as an adjuvant treatment against metastatic breast cancer. NCCN guidelines recommend the combination of capecitabine and docetaxel for recurrent or metastatic breast cancer. Although several PhaseII/III studies have suggested that capecitabine may also be highly active in the neoadjuvant setting, various studies have given conflicting reports of its efficacy in this context. In order to reassess the data already present in the literature with the largest possible statistical power, we carried out what is, to the best of our knowledge, the first meta-analysis of the effects of including capecitabine as part of neoadjuvant polychemotherapy in patients with breast cancer.

Several Phase II trials have suggested that capecitabine holds promise as a component in neoadjuvant chemotherapy, helping patients to achieve high pCR rates (19%–22%) and ORRs (72%–97.6%) [9], [10], [11], [23], [24], [25]in the neoadjuvant setting. Similarly high pCR rates and ORRs were reported in randomized-controlled trials, including the trial by Keun et al [17] and ABCGS24 trial [18]. However, our meta-analysis of five studies showed that including capecitabine in neoadjuvant therapy did not improve pCR in a broad population of stage II/III breast cancer subjects. This result should be interpreted with caution since strong heterogeneity was detected for this outcome across the five studies.

Several factors may explain the lack of capecitabine effect as a part of neoadjuvant therapy. First, all of the patients included in the meta-analysis did not have distant metatasis and had not received previous chemotherapy. The NCCN guideline for these patients recommends treatment with anthracycline and/or taxane chemotherapy which usually proceduces a marked effect. Therefore, the addition of capecitabine to the anthracycline and/or taxane therapies may not show a significant further improvement in clinical efficacy. Second, the capecitabine dosing regimen recommended by NCCN is 1000–1250 mg/m2, twice a day, but several trials included in our meta-analysis reduced this by 14% [14] to 28% [16]. Finally, including capecitabine in the anthracycline and/or taxane therapy resulted in an increase in adverse effects (toxicities), including febrile neutropenia and hand-foot syndrome. The increase in toxicities when capecitabine was included may have led to reduced compliance with therapy.

Although our meta-analysis did not demonstrate an advantage of adding capecitabine to neoadjuvant therapy for breast cancer, some interesting results did emerge from the included studies. The Keun et al trial [17] and ABCSG24 trial [18] reported much higher pCR and ORR when capecitabine was included at a regimen of 1000 mg/m^2^ twice a day. Since this is at the low end of the NCCN recommended dose range, these results suggest that capecitabine can be therapeutically useful at doses that minimize toxic effects. However, this conclusion could not be definitively confirmed because of less data on capecitabine available in the study. The ABCSG24 [18] trial also found that an addition of capecitabine to epirubicin and docetaxel was associated with a greater chance of achieving pCR when the cancer was estrogen receptor negative (ER^−^), not when the cancer was estrogen receptor positive (ER^+^) receiving AT-CMX. Unfortunately we could not perform meta-analysis based on hormone receptor status for lack of sufficient data. Future studies should address this potentially clinically important relationship between capecitabine and hormone receptors.

Our meta-analysis contains some important limitations. First, our study was based on statistical data instead of individual patient data (IPD), which may not provide robust estimation for the association. Second, the characteristics of the included trials were varied in therapy regimen, agents and dosage. The five trials did not use the double-blinding method. Future studies should seek to minimize these possible sources of heterogeneity. Finally, the data from the ABCSG24 trial [18] was available only from a conference abstract and, so complete data has to be expected until publication in a peer-reviewed journal.

Despite the limitations of our research, the results strongly suggest that adding capecitabine to taxane and/or anthracycline neoadjuvant polychemotherapy dose not improve pCR, tnpCR, BCS, or ORR in breast cancer patients without distant metastasis. Further well-designed clinical research with varying drug schedules for the stage II/III breast cancer is required to evaluate the conclusion. The benefits of capecitabine used in neoadjuvant should be balanced against their toxicity, and physicians should take these adverse drug events into consideration and interpret the results carefully and comprehensively in clinical practice.

## Supporting Information

Table S1 **Characteristics of studies included in the meta-analysis.**
(DOC)

Table S2 **Summary of drug-related toxicities grade 3 or greater.**
(DOC)

Checklist S1 **PRISMA checklist.**
(DOC)

## References

[1] van der Hage JA, van de Velde CJ, Julien JP, Tubiana-Hulin M, Vandervelden C, et al (2001) Preoperative chemotherapy in primary operable breast cancer: results from the European Organization for Research and Treatment of Cancer trial 10902. J Clin Oncol 19: 4224–4237. 11709566 10.1200/JCO.2001.19.22.4224

[2] Goldhirsch A, Wood WC, Gelber RD, Coates AS, Thurlimann B, et al (2003) Meeting highlights: updated international expert consensus on the primary therapy of early breast cancer. J Clin Oncol 21: 3357–3365. 12847142 10.1200/JCO.2003.04.576

[3] Koukourakis GV, Kouloulias V, Koukourakis MJ, Zacharias GA, Zabatis H, et al (2008) Efficacy of the oral fluorouracil pro-drug capecitabine in cancer treatment: a review. Molecules 13: 1897–1922. 18794792 10.3390/molecules13081897 PMC6245068

[4] Sawada N, Ishikawa T, Fukase Y, Nishida M, Yoshikubo T, et al (1998) Induction of thymidine phosphorylase activity and enhancement of capecitabine efficacy by taxol/taxotere in human cancer xenografts. Clin Cancer Res 4: 1013–1019. 9563897

[5] Blum JL (2001) The role of capecitabine, an oral, enzymatically activated fluoropyrimidine, in the treatment of metastatic breast cancer. Oncologist 6: 56–64. 11161228 10.1634/theoncologist.6-1-56

[6] O'Shaughnessy J (2002) Clinical experience of capecitabine in metastatic breast cancer. Eur J Cancer 38 Suppl 210–14. 11841930 10.1016/s0959-8049(01)00416-6

[7] Reichardt P, Von Minckwitz G, Thuss-Patience PC, Jonat W, Kolbl H, et al (2003) Multicenter phase II study of oral capecitabine (Xeloda(")) in patients with metastatic breast cancer relapsing after treatment with a taxane-containing therapy. Ann Oncol 14: 1227–1233. 12881384 10.1093/annonc/mdg346

[8] Bevers TB, Anderson BO, Bonaccio E, Buys S, Daly MB, et al (2009) NCCN clinical practice guidelines in oncology: breast cancer screening and diagnosis. J Natl Compr Canc Netw 7: 1060–1096. 19930975 10.6004/jnccn.2009.0070

[9] Lebowitz PF, Eng-Wong J, Swain SM, Berman A, Merino MJ, et al (2004) A phase II trial of neoadjuvant docetaxel and capecitabine for locally advanced breast cancer. Clin Cancer Res 10: 6764–6769. 15501952 10.1158/1078-0432.CCR-04-0976

[10] Manga GP, Shahi PK, Urena MM, Pereira RQ, Plaza MI, et al (2010) Phase II study of neoadjuvant treatment with doxorubicin, docetaxel, and capecitabine (ATX) in locally advanced or inflammatory breast cancer. Breast Cancer 17: 205–211. 19551465 10.1007/s12282-009-0136-6

[11] Jinno H, Sakata M, Hayashida T, Takahashi M, Mukai M, et al (2010) A phase II trial of capecitabine and docetaxel followed by 5-fluorouracil/epirubicin/cyclophosphamide (FEC) as preoperative treatment in women with stage II/III breast cancer. Ann Oncol 21: 1262–1266. 19854722 10.1093/annonc/mdp428

[12] Perez-Manga G, Mendez M, Palomero MI, et al.. (2005) Preliminary results of a phase II study of neoadjuvant treatment with docetaxel (T), doxorubicin(A) and capecitabine (X) in locally advanced or inflammatory breast cancer. EJC Suppl: 133.

[13] Lybaert W, Wildiers H, Neven P, et al.. (2005) Neoadjuvant capecitabine (X) plus docetaxel (T) for patients (pts) with locally advanced breast cancer (LABC): preliminary safety and efficacy data. EJC Suppl: 105.

[14] von MG, Rezai M, Loibl S, Fasching PA, Huober J, et al (2010) Capecitabine in addition to anthracycline- and taxane-based neoadjuvant treatment in patients with primary breast cancer: phase III GeparQuattro study. J Clin Oncol 28: 2015–2023. 20308671 10.1200/JCO.2009.23.8303

[15] Zambetti M, Mansutti M, Gomez P, Lluch A, Dittrich C, et al (2012) Pathological complete response rates following different neoadjuvant chemotherapy regimens for operable breast cancer according to ER status, in two parallel, randomized phase II trials with an adaptive study design (ECTO II). Breast Cancer Res Treat 132: 843–851. 21750964 10.1007/s10549-011-1660-6

[16] Bear HD, Tang G, Rastogi P, Geyer CE Jr, Robidoux A, et al (2012) Bevacizumab added to neoadjuvant chemotherapy for breast cancer. N Engl J Med 366: 310–320. 22276821 10.1056/NEJMoa1111097 PMC3401076

[17] Lee KS, Ro J, Nam BH, Lee ES, Kwon Y, et al (2008) A randomized phase-III trial of docetaxel/capecitabine versus doxorubicin/cyclophosphamide as primary chemotherapy for patients with stage II/III breast cancer. Breast Cancer Res Treat 109: 481–489. 17653851 10.1007/s10549-007-9672-y

[18] Steger GG, Greil R, Jakesz R, Lang A, Mlineritsch B, et al.. (2010) Pathologic complete response (pCR) in patient subgroups: An analysis of ABCSG-24, a phase III, randomized study of anthracycline- and taxane-based neoadjuvant therapy with or without capecitabine in early breast cancer (EBC). J Clin Oncol. June: abstr 530.

[19] Jadad AR, Moore RA, Carroll D, Jenkinson C, Reynolds DJ, et al (1996) Assessing the quality of reports of randomized clinical trials: is blinding necessary. Control Clin Trials 17: 1–12. 8721797 10.1016/0197-2456(95)00134-4

[20] Kuritz SJ, Landis JR, Koch GG (1988) A general overview of Mantel-Haenszel methods: applications and recent developments. Annu Rev Public Health 9: 123–160. 3288229 10.1146/annurev.pu.09.050188.001011

[21] DerSimonian R, Kacker R (2007) Random-effects model for meta-analysis of clinical trials: an update. Contemp Clin Trials 28: 105–114. 16807131 10.1016/j.cct.2006.04.004

[22] Egger M, Davey SG, Schneider M, Minder C (1997) Bias in meta-analysis detected by a simple, graphical test. BMJ 315: 629–634. 9310563 10.1136/bmj.315.7109.629 PMC2127453

[23] Villman K, Ohd JF, Lidbrink E, Malmberg L, Lindh B, et al (2007) A phase II study of epirubicin, cisplatin and capecitabine as neoadjuvant chemotherapy in locally advanced or inflammatory breast cancer. Eur J Cancer 43: 1153–1160. 17398088 10.1016/j.ejca.2007.02.002

[24] Greil R, Moik M, Reitsamer R, Ressler S, Stoll M, et al (2009) Neoadjuvant bevacizumab, docetaxel and capecitabine combination therapy for HER2/neu-negative invasive breast cancer: Efficacy and safety in a phase II pilot study. Eur J Surg Oncol 35: 1048–1054. 19250795 10.1016/j.ejso.2009.01.014

[25] Lu YS, Chen DR, Tseng LM, Yeh DC, Chen ST, et al (2011) Phase II study of docetaxel, capecitabine, and cisplatin as neoadjuvant chemotherapy for locally advanced breast cancer. Cancer Chemother Pharmacol 67: 1257–1263. 20700740 10.1007/s00280-010-1401-2

