# Supplementary material for: Clinical Efficacy of Including Capecitabine in Neoadjuvant Chemotherapy for Breast Cancer: A Systematic Review and Meta-Analysis of Randomized Controlled Trials
Source: PLoS One. 2013 Jan 3;8(1):e53403. doi: 10.1371/journal.pone.0053403 (PMC3536736; doi:10.1371/journal.pone.0053403)
Supplement: Table S1 — Characteristics of studies included in the meta-analysis. (DOC) [file pone.0053403.s001.doc]

Table S1. Characteristics of studies included in the meta-analysis

| Trials | Year | Number of Patients | Therapy of Treatment Arm | Therapy of Control Arm | Age rang  (years) | Clincal T-stage | Clincal N-stage | End-point | Jadad score |
| --- | --- | --- | --- | --- | --- | --- | --- | --- | --- |
| ECTOII[15] | 2010 | 310 | A 60 mg/m2 + T 200 mg/m2 *4 followed by C 600 mg/m2 + M 40mg/m2 + X 925 mg/m2 *4 A 60 mg/m2 + C 600 mg/m2 *4 followed by T 100 mg/m2 + X 925 mg/m2 *4 | A 60 mg/m2 + T 200mg/m2 *4 followed by C 600 mg/m2 + M 40 mg/m2 + F 600 mg/m2 *4 | 22-77 | NR | N0-N3 | pCR,tnpCR, ORR | 3 |
|  |  |  |  |  | 29-71 |  |  |  |  |
| Korean[17] | 2008 | 209 | T 75 mg/m2 + X 1000 mg/m2 *4 | A 60 mg/m2 + C 600 mg/m2 *4 | 24-67 | T1-T4 | N1-N3 | pCR, tnpCR, ORR, BCS | 3 |
|  |  |  |  |  | 21-65 |  |  |  |  |
| NSABP B-40[16] | 2012 | 805 | T 75 mg/m2 + X 825 mg/m2 *4 followed by A 60 mg/m2 + C 600 mg/m2 *4 | T 100 mg/m2 *4 followed by A 60 mg/m2 + C 600 mg/m2 *4 | NR | NR | NR | pCR, tnpCR, ORR, BCS | 3 |
|  |  |  |  |  | NR |  |  |  |  |
| GEPARQUATTRO[14] | 2012 | 1421 | E 90 mg/m2 + C 600 mg/m2 *4 followed by T 75 mg/m2 + X 900 mg/m2 *4 E 90 mg/m2 + C 600 mg/m2 *4 followed by T 75 mg/m2 *4 followed by X 900 mg/m2 *4 | E 90 mg/m2 + C 600 mg/m2 *4 followed by T 100 mg/m2 *4 | 23-78 | T1-T4 | N0-N3 | pCR, tnpCR, ORR, BCS | 3 |
|  |  |  |  |  | 22-75 |  |  |  |  |
| ABCSG24[18] | 2010 | 512 | E 75 mg/m2 + T 75 mg/m2 + X 1000 mg/m2 *6 | E 75 mg/m2 + T 75 mg/m2 *6 | 25-71 | T1-T4 | NR | pCR, ,BCS | 3 |
|  |  |  |  |  | 27-73 |  |  |  |  |

Abbreviations: A : doxorubicin; C : cyclophosphamide ; T: docetaxel; E : epirubicin; F : fluorouracil; M : methotrexate; X : capecitabine; NR: not reported; pCR : pathological complete responses in breast; tnpCR: pCR in breast tumor and nodes; ORR: overall response rate; BCS: breast-conserving surgery
