# Supplementary material for: Clinical Efficacy of Including Capecitabine in Neoadjuvant Chemotherapy for Breast Cancer: A Systematic Review and Meta-Analysis of Randomized Controlled Trials
Source: PLoS One. 2013 Jan 3;8(1):e53403. doi: 10.1371/journal.pone.0053403 (PMC3536736; doi:10.1371/journal.pone.0053403)
Supplement: Table S2 — Summary of drug-related toxicities grade 3 or greater. (DOC) [file pone.0053403.s002.doc]

Table S2. Summary of drug-related toxicities grade 3 or greater

| Adverse events | No. of studies[14, 16, 17] | No. of patients | Heterogeneity | | Statistical model | Effect size | | |
| --- | --- | --- | --- | --- | --- | --- | --- | --- |
|  |  |  | p | I^2^ (%) |  | OR | 95%CI | p |
| Febrile neutropenia | 3 | 2453 | 0.58 | 0 | Fixed- effect model | 1.55 | 1.11 – 2.15 | 0.01 |
| Hand-foot syndrome | 3 | 2453 | 0.004 | 82 | Random-effect model | 7.26 | 2.35 – 22.43 | <0.01 |
| Neutropenia | 3 | 2453 | 0.01 | 77 | Random-effect model | 0.95 | 0.58 – 1.55 | 0.84 |
| Vomiting | 3 | 2453 | <0.01 | 97 | Random-effect model | 0.32 | 0.02 – 6.51 | 0.46 |
